# Human infection and environmental contamination with Avian Influenza A (H7N9) Virus in Zhejiang Province, China: risk trend across the three waves of infection

**DOI:** 10.1186/s12889-015-2278-0

**Published:** 2015-09-21

**Authors:** Fan He, En-Fu Chen, Fu-Dong Li, Xin-Yi Wang, Xiao-Xiao Wang, Jun-Fen Lin

**Affiliations:** Zhejiang Provincial Center for Disease Control and Prevention, 3399 Binsheng Road, Binjiang District, Hangzhou, Zhejiang 310051 People’s Republic of China

**Keywords:** Avian Influenza A (H7N9) Virus, Surveillance, Live poultry markets

## Abstract

**Background:**

The third wave of H7N9 cases in China emerged in the second half of 2014. This study was conducted to identify the risk trends of H7N9 virus in human infections and environment contamination.

**Methods:**

A surveillance program for H7N9 virus has been conducted in all 90 counties in Zhejiang since March 2013. All H7N9 cases were reported by hospitals through the China Information System for Disease Control and Prevention. Sampling sites for environment specimens were randomly selected by a multi-stage sampling strategy. Poultry-related workers for serological surveillance were randomly selected from the sampling sites for environmental specimens in the first quarter of each year. rRT-PCR and viral isolation were performed to identify H7N9 virus. A hemagglutination inhibition assay was conducted to detect possible H7N9 infection among poultry-related workers.

**Results:**

A total of 170 H7N9 cases were identified in Zhejiang from 20 March 2013 to 28 February 2015. The proportion of rural cases increased from 42.2 % (19/45) to 67.7 % (21/31) with progression of the three epidemics (P < 0.05). In 32 % (161/503) of towns and 16.0 % (238/1488) of surveyed premises, H7N9 virus was detected in the environment. The positive rate of environmental specimens was 6.1 % (868/14207). In addition, 912 poultry-related workers were recruited and 3.7 % (34) of them tested positive for H7N9 antibodies. Positive detection of H7N9 virus during environmental surveillance increased from the first to third wave (P < 0.05). Almost all positive rates of environmental surveillance were higher in urban than rural in the second wave (P < 0.05), however they were higher in rural area in the third wave (P < 0.05).

**Conclusions:**

Our study highlights that the severity of poultry-related environmental contamination by H7N9 virus is intensifying. We strongly recommend that the local government stop illegal trading immediately and close live poultry markets in the territory. Poultry operations in slaughtering plants must be supervised rigorously. Prior to the closure of live poultry markets, daily cleaning and disinfecting of areas potentially contaminated by H7N9 virus, centralized collection and disposal of trash, designating certain days as market rest days, banning overnight poultry storage and other measures should be strictly carried out in both urban and rural areas.

## Background

Since a novel reassortant avian influenza A (H7N9) virus emerged in China in February 2013 [1], China experienced two waves of H7N9 human infection that resulted in over 400 human cases [2, 3]. The third wave emerged on 1 November 2014, and nearly 200 H7N9 human infections have been identified as of 28 February 2015.

Earlier studies suggested that the H7N9 virus is a multiple reassortant virus, with gene fragments derived from H7N9, H9N2 and H7N3 subtypes of influenza A virus [4, 5]. Poultry farms and live poultry markets (LPMs) are possible sources of reassortant virus and human infection [6–8].

Zhejiang is a province in southeastern China with the second highest number of H7N9 cases. It consists of 11 prefectures and 90 counties and has a population of more than 50 million. It is one of the most developed provinces in China, with large-scale poultry and swine breeding industries. Since the first H7N9 case emerged in March 2013 in Zhejiang Province, virologic surveillance for H7N9 virus has been conducted in LPMs, poultry farms, slaughtering and processing plants, and habitats for migratory birds within the province.

The past two epidemics led to 139 H7N9 cases (49 deaths) in Zhejiang Province, of which 72 (52 %) subjects were urban residents. The government closed the LPMs in the central towns, and the epidemic was controlled effectively (77 cases occurred before and 17 cases after market closures during the second wave). Compared to over half of cases distributed in urban areas in the past two waves, all 14 of the first cases in the third wave were infected in rural LPMs. This suggests new characteristics of H7N9 virus. The aim of this study was to identify the epidemiological characteristics and risk trend of H7N9 virus in human infection, environmental contamination and incidence of infection of poultry-related workers.

## Methods

### Definition of the three waves

Based on the date of onset, the first wave in Zhejiang Province was defined from 20 March to 30 September 2013. In this wave, the first and last cases occurred on 20 March and 16 April 2013, respectively. No additional cases were identified until October 2013. We defined the second wave as starting from 1 October 2013 and ceasing on 31 October 2014. In the second wave, few cases were reported after March 2014 except for one person who developed symptoms on June 3, 2014. The third wave started on 1 November 2014 and continues as of the date of writing.

### Confirmation of patients

A confirmed case is defined as clinical symptoms consistent with acute influenza (fever, cough, coryza, difficulty breathing) or with a history of contact with a confirmed or suspected case and a laboratory test positive for avian influenza A (H7N9) virus, with subtype confirmed by PCR, viral isolation or a four-fold or greater increase in serum antibodies specific for this virus isolated from paired sera. A severe case is a confirmed case with pneumonia complicated by respiratory failure or other organ failure [9].

The surveillance protocol for H7N9 cases stipulates that all hospitals in Zhejiang Province must report all cases through the China Information System for Disease Control and Prevention [10]. Respiratory specimens are to be collected from patients with influenza and tested for H7N9 viral nucleic acid by hospitals or local influenza network laboratories. Positive specimens are to be forwarded to the provincial influenza network laboratory for viral isolation. Serum samples collected by hospitals are to be sent through the local influenza network laboratory to the provincial influenza network laboratory for antibody testing.

### Surveillance sites and sample collection

A surveillance program on H7N9 virus was conducted from March 2013 to February 2015 in all 90 counties of the 11 prefectures in Zhejiang Province. A multi-stage sampling strategy was used to select sampling sites for collection of environmental specimens. In each prefecture, one-third of counties were surveyed per quarter and all counties would have been covered in 2013. In 2014, the frequency was changed to monthly with all counties covered quarterly. Then, two or more premises were randomly selected in each county and at least two sampling sites were randomly selected at each of the premises. About 10 environmental specimens (1–2 for each type of sample) were collected from each sampling site. Overall, each prefecture was asked to collect 15–30 specimens each time for every type of environmental sample, which added up to at least 30 specimens during the influenza epidemic period (October to March of the following year).

The environmental sampling premises included LPMs, poultry rearing farms, concentrated areas of backyard poultry farms, slaughtering and processing plants, habitats for migratory birds, and other poultry-related premises (such as living quarters of H7N9 cases, restaurants, supermarkets, and markets selling cold fresh poultry). The sampling premises were randomly selected in the area and had to meet one of the following criteria: (1) had reported human or poultry infection with H7N9 virus; (2) had a relatively high density of lakes, rivers or other bodies of water; (3) had a developed poultry breeding industry; (4) was near habitat for migratory birds or was located on a migration route; (5) had a high proportion of domestic poultry backyard farmers.

A sampling site was defined as a stall in the LPMs, a poultry housing in the poultry rearing farms, a raising-household in the concentrated areas of backyard poultry farms and a random site at different direction in the habitats for migratory birds.

Each type of environmental sample was collected prior to disinfection, including fecal dropping swabs, poultry cage swabs, drinking water samples, sewage from cleaning poultry, and swabs of tables used for slaughtering or sprocessing poultry. Fecal specimens of 3–5 g were collected from fresh fecal droppings and put into sampling tubes. Poultry cage specimens were collected through swabbing 3–5 positions on the cage surface where frequently touched by poultry. Drinking water specimens of 5–10 ml were collected from water troughs that were shared by all the poultry in the same cage. Sewage (5–10 ml) was gathered from basins or buckets used for cleaning poultry. The method of collecting specimens from tables for slaughtering or processing poultry was the same as for collecting poultry cage specimens. All environmental specimens were disrupted repeatedly in sterile conditions to break up the solid matter, and then were precipitated for 30 min at 4 °C or centrifuged for 10 min at 3000 rpm. The supernatant was divided into three aliquots for detection, preservation or validation.

All environmental samples were stored at 4 °C and transported to local influenza network laboratories at the 11 prefectural centers for disease control and prevention (CDCs) within 48 h. Then, each sample was divided equally into three parts and stored at −70 °C. The first part was tested for influenza A viral nucleic acid in the network laboratory within one week. Samples positive for influenza A virus were further typed as H7, H9 and N9. For those samples positive for influenza A virus, another two parts were transported to the Zhejiang provincial influenza network laboratory for validation and preservation.

Serological surveillance among poultry-related workers was performed in the first quarter of each year. The poultry-related workers were randomly selected from the sampling sites for collection of environmental specimens in the first quarter of 2013 and 2014, whether they were healthy or not. At least two workers were surveyed at every sampling site, and each prefecture was asked to collect at least 35 samples per year. The surveillance subjects were those who directly contacted poultry (physical contact with poultry or related biological matter, including blood, internal organs, eggs, secretions, feces, or poultry cages) [11] in LPMs (wholesale and retail), poultry rearing farms, backyard poultry farms, slaughtering and processing plants, and those who directly contacted migratory birds or their excreta in habitats. Eligible workers were identified through inquiring about their work in detail and from field observation. Fasting venous blood (5 ml) was collected from each worker. Serum samples were prepared by precipitation and centrifugation using standard procedures and then divided into three aliquots. The first was preserved in network laboratories of the 11 prefectural CDCs at −20 °C or below. The other two were transported to Zhejiang Provincial CDC (one for detecting antibodies against influenza A (H7N9) virus and the other for transport to the Chinese National Influenza Center for validation).

### Statistical analysis

We compared the age of H7N9 cases and that of poultry-related workers between the three waves using Student’s *t*-test or F test. Pearson’s chi-square or Fisher’s exact test were used to compare sex and occupational distribution of the cases between the three waves, and to compare the positive rates between urban (including cities and suburbs) and rural areas (referring to the countryside). A trend chi-square test was used to analyze the trend of positive rates across the three waves.

### Laboratory testing

Respiratory specimens of the cases were tested by rRT-PCR and viral isolation following standard laboratory protocols [9]. RNA was extracted from specimens with the Qiagen RNeasy mini kit, and rRT-PCR was then performed with H7N9-specific primers and probes as per the manufacturer’s protocol. For viral isolation, we inoculated respiratory specimens in allantoic cavities of pathogen-free embryonated chicken eggs. Environmental specimens were also tested by rRT-PCR using the same protocols described above. For fasting serum specimens from poultry workers, antibodies against H7N9 virus were detected by a hemagglutination inhibition assay using horse red blood cells, following laboratory procedures issued by the World Health Organization [12]. rRT-PCR was performed at local influenza network laboratories and validated by the Zhejiang provincial influenza network laboratory. The hemagglutination inhibition assay was conducted at laboratory of Zhejiang provincial influenza network laboratory and validated by Chinese National Influenza Center.

### Ethical review

Our study was reviewed and approved by the Ethics Committee of the Zhejiang Provincial Center for Disease Control and Prevention. All poultry-related workers provided written consents of participating in the study before investigators starting the interviews.

## Results

### Distribution of H7N9 human infections

From March 2013 to February 2015, a total of 170 H7N9 human cases were identified in Zhejiang Province, China. In each respective wave, 45, 94 and 31 cases were found, which were distributed in 35, 79 and 30 towns; 17, 42 and 23 counties; and 5, 9 and 8 prefectures.

Of the 170 cases, 51.8 % (88/170) were from rural areas. The proportion of rural cases increased from 42.2 to 67.7 % across the three waves (trend *χ*^2^ = 4.549, *P* = 0.038). About 19 % cases were identified after the LPMs were closed and the proportion increased from 11 % in the first wave to 32 % in the third wave (trend *χ*^2^ = 5.067, *P* = 0.027). There was no significant upward or downward trend in death rate among the three waves (trend *χ*^2^ = 3.767, *P* = 0.052). The mean age of H7N9 cases was 59.7 ± 14.4 years in the first wave, 55.3 ± 17.5 years in the second wave and 55.7 ± 13.6 years in the third wave (*F* = 1.170, *P* = 0.313). The sex distribution did not differ significantly between the first wave (62.2 % males), second wave (68.1 % males) and third wave (71.0 % males) (*χ*^2^ = 0.736, *P* = 0.699). Although no significant difference was seen among the three waves in occupational distribution of H7N9 cases, there were more farmers in the third wave (64.5 %) than in the first (37.8 %) and second wave (46.8 %) (Table 1).

**Table 1:** Characteristics of H7N9 human infections by wave of infection— Zhejiang Province, China, March 2013-February 2015

Characteristics	Total (*n* = 170)	Wave				
		First (*n* = 45)	Second (*n* = 94)	Third (*n* = 31)	Statistic	P value
Rural, n (%)	88(51.8)	19(42.2)	48(51.1)	21(67.7)	4.549	0.038
Onset after closure of live poultry markets, n (%)	32(18.8)	5(11.1)	17(18.1)	10(32.3)	5.067	0.027
Deaths, n (%)	56(32.9)	10(22.2)	39(41.5)	13(41.9)	3.767	0.052
Age (years)	56.5 ± 16.1	59.7 ± 14.4	55.3 ± 17.5	55.7 ± 13.6	1.170	0.313
Male, n (%)	114(67.1)	28(62.2)	64(68.1)	22(71.0)	0.736	0.699
Occupation, n (%)					16.001	0.068
Farmer	81(47.6)	17(37.8)	44(46.8)	20(64.5)		
Homemaker	11(6.5)	4(8.9)	3(3.2)	4(12.9)		
Retiree	37(21.8)	14(31.1)	18(19.1)	5(16.1)		
Self-employed	22(12.9)	6(13.3)	14(14.9)	2(6.5)		
Worker	16(9.4)	4(8.9)	12(12.8)	0(0.0)		
Child	3(1.8)	0(0.0)	3(3.2)	0(0.0)		

**Fig. 1:**
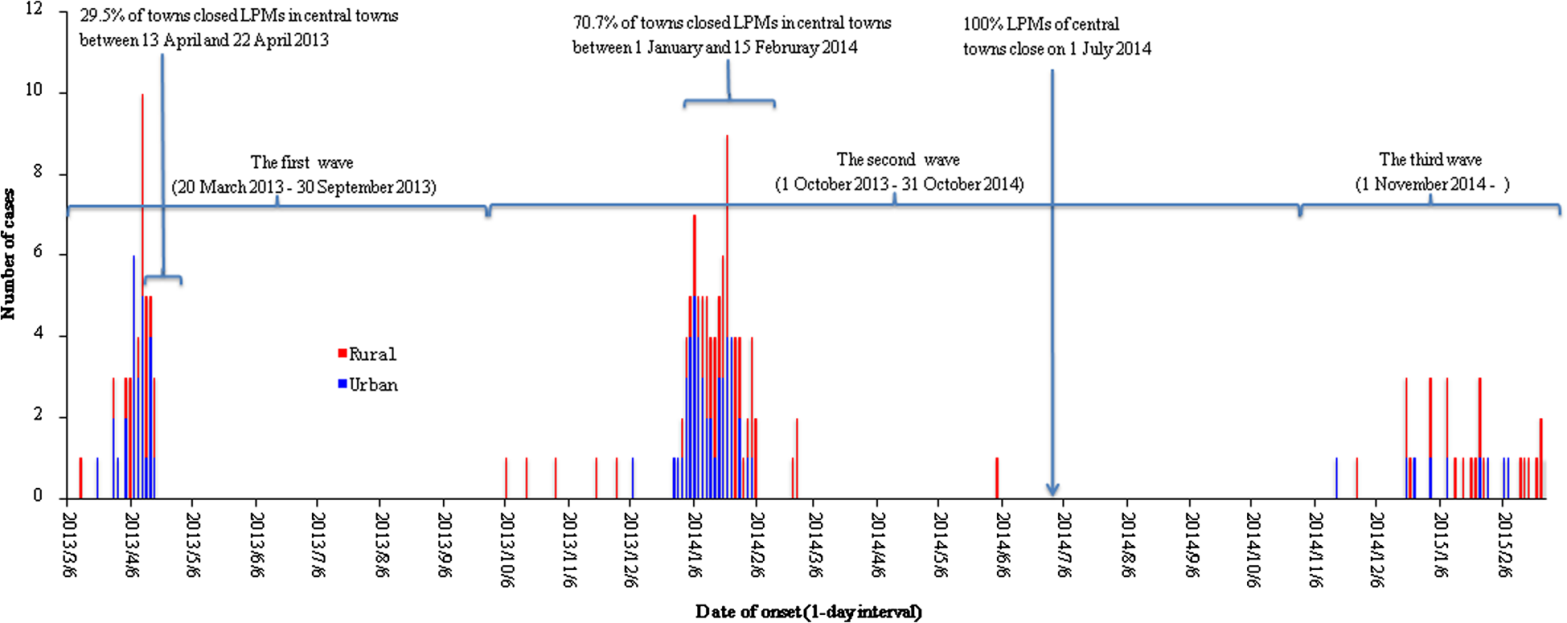
Epidemic curve for H7N9 human infections— Zhejiang Province, China, March 2013-February 2015 at 1-day intervals

Since the first H7N9 case emerged on 13 March 2013 in the first wave, the number of cases increased sharply and most occurred in the first half of April 2013. The last case of this wave occurred on 16 April 2013. Of all 1,329 towns in Zhejiang Province, 29.5 % (including all towns with case reports) closed the LPMs of central towns between 13 and 22 April 2013. The first case in the second wave emerged on 6 October 2013. The onset dates of most cases were concentrated in January of 2014. After 70.7 % of towns closed LPMs in town centers between 1 January and 15 February in 2014, the number of cases declined rapidly. Although the Zhejiang provincial government ordered closing all LPMs of central towns on 1 July 2014, a new H7N9 human case emerged in November of 2014, marking the beginning of the ongoing third wave (Fig. 1).

### Risk of H7N9 virus in poultry-related environments

A total of 1,488 poultry-related premises were selected as surveillance sites from March 2013 to February 2015, which were distributed in 503 towns of 90 counties in Zhejiang Province. Of the 1,488 premises, 63.1 % (939), 15.8 % (235), 11.6 % (173), 2.4 % (36), 2.2 % (32) and 4.9 % (73) were LPMs, poultry rearing farms, concentrated areas of backyard poultry farms, slaughtering and processing plants, habitats for migratory birds, and other premises, respectively.

We collected 14,207 environmental specimens from the premises, and 6.1 % (868) tested positive for H7N9 virus. The positive rates in LPMs (8.8 %) and other poultry-related premises (8.2 %) were higher than in slaughtering and processing plants (4.9 %), poultry rearing farms (0.3 %), concentrated areas of backyard poultry farms (0.1 %), and habitats for migratory birds (0.0 %) (*χ*^2^ = 549.351, *P* < 0.001). Overall, the positive rates of H7N9 virus in the environment presented a gradually increasing trend in Zhejiang Province during the period March 2013- February 2015. There were two peaks, in January of 2014 and 2015 (Table 2).

**Table 2:** Monthly results of surveillance for H7N9 virus at different premises of Zhejiang Province, China, March 2013-February 2015

Month	Positive rate, % (positive specimens/total specimens)						
	Total	Live poultry markets	Poultry rearing farms	Concentrated areas of backyard poultry farms	Slaughtering and processing plants	Habitats for migratory birds	Others
2013–3	0.0 (0/119)	0.0 (0/77)	0.0 (0/24)	0.0 (0/10)	0/0	0/0	0.0 (0/8)
2013–4	1.1 (11/1045)	2.3 (7/303)	1.0 (4/382)	0.0 (0/201)	0.0 (0/17)	0.0 (0/59)	0.0 (0/83)
2013–5	0.0 (0/96)	0/0	0.0 (0/46)	0.0 (0/30)	0/0	0.0 (0/20)	0/0
2013–6	0.0 (0/60)	0.0 (0/19)	0.0 (0/35)	0.0 (0/6)	0/0	0/0	0/0
2013–7	0.0 (0/57)	0.0 (0/20)	0.0 (0/31)	0/0	0/0	0/0	0.0 (0/6)
2013–8	0.0 (0/62)	0.0 (0/43)	0.0 (0/12)	0/0	0/0	0.0 (0/7)	0/0
2013–9	0.0 (0/60)	0.0 (0/49)	0.0 (0/11)	0/0	0/0	0/0	0/0
Total of the 1st wave	0.7 (11/1499)	1.4 (7/511)	0.7 (4/541)	0.0 (0/247)	0.0 (0/17)	0.0 (0/86)	0.0 (0/97)
2013–10	2 (6/301)	3.1 (6/195)	0.0 (0/75)	0.0 (0/25)	0/0	0/0	0.0 (0/6)
2013–11	1.1 (4/372)	1.5 (4/266)	0.0 (0/62)	0.0 (0/19)	0/0	0.0 (0/5)	0.0 (0/20)
2013–12	3.5 (11/318)	4.2 (11/262)	0.0 (0/30)	0/0	0.0 (0/8)	0.0 (0/5)	0.0 (0/13)
2014–1	10.2 (259/2551)	11.3 (259/2299)	0.0 (0/105)	0.0 (0/133)	0.0 (0/2)	0.0 (0/9)	0.0 (0/3)
2014–2	2.7 (29/1073)	4.0 (29/732)	0.0 (0/141)	0.0 (0/146)	0.0 (0/6)	0/0	0.0 (0/48)
2014–3	1.6 (9/554)	3.7 (9/242)	0.0 (0/146)	0.0 (0/114)	0.0 (0/18)	0.0 (0/5)	0.0 (0/29)
2014–4	1.1 (6/545)	3.1 (6/194)	0.0 (0/168)	0.0 (0/164)	0.0 (0/5)	0.0 (0/14)	0/0
2014–5	0.3 (2/714)	0.7 (2/303)	0.0 (0/83)	0.0 (0/279)	0.0 (0/5)	0.0 (0/14)	0.0 (0/30)
2014–6	3.4 (10/290)	6.8 (8/118)	2.6 (2/78)	0.0 (0/53)	0.0 (0/25)	0.0 (0/16)	0/0
2014–7	1.7 (6/358)	2.7 (6/219)	0.0 (0/72)	0.0 (0/22)	0.0 (0/10)	0.0 (0/9)	0.0 (0/26)
2014–8	0.0 (0/519)	0.0 (0/298)	0.0 (0/104)	0.0 (0/58)	0.0 (0/32)	0.0 (0/24)	0.0 (0/3)
2014–9	2.1 (9/419)	3.8 (9/234)	0.0 (0/80)	0.0 (0/43)	0.0 (0/18)	0.0 (0/22)	0.0 (0/22)
2014–10	3.9 (25/633)	5.9 (25/424)	0.0 (0/119)	0.0 (0/18)	0.0 (0/12)	0.0 (0/11)	0.0 (0/49)
Total of the 2nd wave	4.3 (376/8647)	6.5 (374/5786)	0.2 (2/1263)	0.0 (0/1074)	0.0 (0/141)	0.0 (0/134)	0.0 (0/249)
2014–11	3(17/572)	5.0 (17/343)	0.0 (0/69)	0.0 (0/18)	0.0 (0/62)	0.0 (0/45)	0.0 (0/35)
2014–12	2.6(21/808)	3.8 (19/499)	0.0 (0/142)	0.0 (0/70)	8.0 (2/25)	0.0 (0/44)	0.0 (0/28)
2015–1	20.2(242/1197)	24.3 (198/815)	0.0 (0/151)	0.0 (0/32)	17.8 (13/73)	0.0 (0/6)	25.8 (31/120)
2015–2	13.5(201/1484)	16.8 (170/1012)	0.0 (0/127)	1.5 (2/130)	7.7 (2/26)	0.0 (0/8)	14.9 (27/181)
Total of the 3rd wave	11.8 (481/4061)	15.1 (404/2669)	0.0 (0/489)	0.8 (2/250)	9.1 (17/186)	0.0 (0/103)	15.9 (58/364)
Total	6.1(868/14207)	8.8 (785/8966)	0.3 (6/2293)	0.1 (2/1571)	4.9 (17/344)	0.0 (0/323)	8.2 (58/710)

Of 503 selected towns, 32.0 % (161) had H7N9 virus detected in their environment. The proportion of positive towns was 5.6 % in the first wave, which went up to 19.5 % in the second wave and 37.1 % in the third wave (trend *χ*^2^ = 45.996, *P* < 0.001). At the same time, 16.0 % (238/1,488) of premises tested positive for H7N9 virus and this proportion increased from 2.8 to 28.2 % across the three waves trend *χ*^2^ = 91.319, *P* < 0.001). The positive rates of H7N9 virus in environmental specimens in the three waves of infection increased gradually from 0.7 to 11.8 % (trend *χ*^2^ = 335.989, *P* < 0.001). Moreover, all positive rates of H7N9 virus from the various premises and samples showed significant upward trends during the progress of the three waves (all P values less than 0.05) (Table 3).

**Table 3:** Comparison on positive rates of environmental surveillance for H7N9 virus, by three waves — Zhejiang Province, China, March 2013-February 2015

Characteristics	1st wave	2nd wave	3rd wave	Total	Trend *χ* ^2^ value	P value
Towns, % (positive towns/total towns)	5.6 (5/90)	19.5 (69/354)	37.1 (104/280)	32.0 (161/503)	45.996	<0.001
Premises, % (positive premises/total premises)	2.8 (5/181)	11.0 (98/890)	28.2 (138/490)	16.0 (238/1488)	91.319	<0.001
Environmental samples of different premises, % (positive specimens/total specimens)	0.7 (11/1499)	4.3 (376/8647)	11.8 (481/4061)	6.1 (868/14207)	335.989	<0.001
Live poultry markets	1.4 (7/511)	6.5 (374/5786)	15.1 (404/2669)	8.8 (785/8966)	203.645	<0.001
Poultry rearing farms	0.7 (4/541)	0.2 (2/1263)	0.0 (0/489)	0.3 (6/2293)	5.558	0.029
Concentrated areas of backyard poultry farms	0.0 (0/247)	0.0 (0/1074)	0.8 (2/250)	0.1 (2/1571)	6.302	0.049
Slaughtering and processing plants	0.0 (0/17)	0.0 (0/141)	9.1 (17/186)	4.9 (17/344)	13.232	<0.001
Habitats for migratory birds	0.0 (0/86)	0.0 (0/134)	0.0 (0/103)	0.0 (0/323)	-	-
Others	0.0 (0/97)	0.0 (0/249)	15.9 (58/364)	8.2 (58/710)	48.346	<0.001
Environment samples of different types, % (positive specimens/total specimens)	0.7 (11/1499)	4.3 (376/8647)	11.8 (481/4061)	6.1 (868/14207)	335.989	<0.001
Fecal dropping swab	0.3 (2/702)	2.8 (94/3306)	9.7 (174/1786)	4.7 (270/5794)	145.342	<0.001
Poultry cage swabs	0.0 (0/315)	5.4 (106/1956)	11.3 (85/750)	6.3 (191/3021)	55.669	<0.001
Drinking water samples	1.0 (2/201)	2.9 (27/943)	10.1 (34/338)	4.3 (63/1482)	31.962	<0.001
Sewage from cleaning poultry	1.5 (1/67)	5.0 (43/859)	20.2 (61/302)	8.6 (105/1228)	62.802	<0.001
Swabs of tables for slaughtering or processing poultry	1.6 (1/61)	7.3 (78/1063)	14.6 (93/636)	9.8 (172/1760)	28.542	<0.001
Others	3.3 (5/153)	5.4 (28/520)	13.7 (34/249)	7.3 (67/922)	18.342	<0.001

Overall, there were no significant differences between urban and rural areas in proportions of H7N9 virus-positive towns (urban: 33.7 %, rural: 31.0 %, *P* = 0.543), premises (urban: 15.4 %, rural: 16.3 %, *P* = 0.711) and environmental specimens (urban: 5.9 %, rural: 6.0 %, *P* = 0.730). All three proportions increased gradually from the first to third wave in both urban and rural areas (*P* < 0.05). Almost no significant differences in proportion of positive H7N9 virus were found between urban and rural areas in the first wave (*P* > 0.05). However, these proportions were higher in urban than in rural areas in the second wave (*P* < 0.05) and conversely, were significantly higher in rural areas in the third wave (*P* < 0.05) (Table 4).

**Table 4:** Comparison of positive rates of environmental surveillance for H7N9 virus by residential area (urban and rural) — Zhejiang Province, China, March 2013-February 2015

Characteristics	1st wave			2nd wave			3rd wave			Total			P value of trend	
	Urban	Rural	P value	Urban	Rural	P value	Urban	Rural	P value	Urban	Rural	P value	Urban	Rural
Towns, % (positive towns/total towns)	2.9 (1/35)	7.3 (4/55)	0.645	25.4 (35/138)	15.6 (34/218)	0.028	29.1 (23/79)	40.3 (81/201)	0.099	33.7 (56/166)	31.0 (105/339)	0.543	0.008	<0.001
Premises, % (positive premises/total premises)	1.7 (1/60)	3.7 (4/108)	0.656	13.7 (49/358)	8.5 (42/497)	0.018	21.2 (35/165)	32.2 (101/314)	0.014	15.4 (85/551)	16.3 (145/889)	0.711	<0.001	<0.001
Environment samples of different premises, % positive specimens/ total specimens)	0.6 (3/472)	0.9 (8/879)	0.756	5.8 (188/3221)	2.1 (94/4444)	<0.001	8.0 (97/1207)	14.2 (382/2697)	<0.001	5.9 (288/4900)	6.0 (484/8020)	0.730	<0.001	<0.001
Live poultry markets	1.2 (3/252)	1.7 (4/229)	0.714	7.1 (188/2637)	3.8 (92/2427)	<0.001	8.7 (59/675)	18.1 (343/1894)	<0.001	7.0 (250/3564)	9.6 (439/4550)	<0.001	<0.001	<0.001
Poultry rearing farms	0.0 (0/128)	1.2 (4/337)	0.579	0.0 (0/184)	0.2 (2/944)	1.000	0.0 (0/165)	0.0 (0/287)	-	0.0 (0/477)	0.4 (6/1568)	0.346	-	0.019
Concentrated areas of backyard poultry farms	0.0 (0/15)	0.0 (0/200)	-	0.0 (0/157)	0.0 (0/891)	-	0.0 (0/60)	1.1 (2/186)	1.000	0.0 (0/232)	0.2 (2/1277)	1.000	-	0.017
Slaughtering and processing plants	0.0 (0/11)	0.0 (0/6)	-	0.0 (0/77)	0.0 (0/44)	-	9.2 (12/131)	9.1 (5/55)	1.000	5.5 (12/219)	4.8 (5/105)	1.000	0.010	0.057
Habitats for migratory birds	0.0 (0/19)	0.0 (0/67)	-	0.0 (0/14)	0.0 (0/84)	-	0.0 (0/6)	0.0 (0/81)	-	0.0 (0/39)	0.0 (0/232)	-	-	-
Others	0.0 (0/47)	0.0 (0/40)	-	0.0 (0/152)	0.0 (0/54)	-	15.3 (26/170)	16.5 (32/194)	0.776	7.0 (26/369)	11.1 (32/288)	0.073	<0.001	<0.001
Environment samples of different types, % (positive specimens/total specimens)	0.6 (3/472)	0.9 (8/879)	0.756	5.8 (188/3221)	2.1 (94/4444)	<0.001	8.0 (97/1207)	14.2 (382/2697)	<0.001	5.9 (288/4900)	6.0 (484/8020)	0.730	<0.001	<0.001
Fecal dropping swab	0.0 (0/190)	0.5 (2/432)	1.000	2.7 (26/977)	1.7 (33/1897)	0.126	4.1 (17/410)	12.3 (156/1273)	<0.001	2.7 (43/1577)	5.3 (191/3602)	<0.001	0.004	<0.001
Poultry cage swab	0.0 (0/114)	0.0 (0/167)	-	7.6 (60/786)	2.0 (19/970)	<0.001	8.7 (17/196)	12.4 (67/540)	0.190	7.0 (77/1096)	5.1 (86/1677)	0.039	0.013	<0.001
Drinking water sample	0.0 (0/47)	1.5 (2/131)	1.000	5.5 (16/289)	1.2 (7/578)	<0.001	5.9 (6/102)	12.8 (28/219)	0.079	5.0 (22/438)	4.0 (37/928)	0.394	0.251	<0.001
Sewage of cleaning poultry	0.0 (0/30)	2.7 (1/37)	1.000	6.3 (25/400)	3.5 (14/395)	0.100	14.0 (18/129)	26.2 (43/164)	0.013	7.7 (43/559)	9.7 (58/596)	0.252	0.001	<0.001
Swab of tables for slaughtering or processing poultry	2.9 (1/34)	0.0 (0/23)	1.000	7.7 (39/509)	4.0 (15/375)	0.032	9.0 (20/223)	18.1 (73/403)	0.002	7.8 (60/766)	11.0 (88/801)	0.038	0.305	<0.001
Others	3.5 (2/57)	3.4 (3/89)	1.000	8.5 (22/260)	2.6 (6/229)	0.006	12.9 (19/147)	15.3 (15/98)	0.706	9.3 (43/464)	5.8 (24/416)	0.056	0.029	0.001

### Serological survey of H7N9 antibodies in poultry-related workers

A total of 912 poultry-related workers were recruited from 337 premises of 75 towns. The mean age was 49.8 ± 11.3 years (range 4–84 years) and 58.2 % of them were male. There were no differences in age (t = −1.363, *P* = 0.173) or sex distribution (*χ*^2^ = 2.364, *P* = 0.124) between the first and second wave.

Of the poultry-related workers, 3.7 % (34) tested positive for H7N9 antibody according to the criteria described in the methods. The positive rate in the second wave (6.5 %, 33/511) was significantly higher than in the first wave (0.25 %, 1/401) (*P* < 0.001). Poultry-related workers from concentrated areas of backyard poultry farms (7.7 %, 22/284) and poultry rearing farms (3.7 %, 11/300) had higher positive rates for H7N9 antibody than those from LPMs (0.4 %, 1/227). No workers with positive H7N9 antibodies were identified from slaughtering and processing plants (0/28), habitats for migratory birds (0/26) or other premises (0/47). Only one worker from an LPM tested positive for H7N9 antibodies in the first wave; the remaining 33 were identified in the second wave (11 from poultry rearing farms and 22 from concentrated areas of backyard poultry farms). In addition, none of the workers reported any respiratory symptoms during the past month.

## Discussion

LPMs are considered to be the sources of H7N9 viral human infections based on the evidence of case exposure history [13–15] and gene sequence similarity between viral isolates [7, 16]. Closure of LPMs was conducted to block the transmission of H7N9 virus and was thought to be the most effective method for restricting the epidemic at the present stage in China [17, 18]. The government of Zhejiang Province closed all LPMs in central towns in July 2014, prior to the emergence of the third wave of infection. The number of H7N9 cases in the third wave was significantly lower than in the previous two waves. However, the closure of LPMs in central towns forced the forwarding of contaminated poultry to rural markets, which led to a relative increase of H7N9 cases in rural areas and an increase in number of infected rural towns. In Zhejiang Province, almost 20 % of cases occurred after closure of LPMs in their residential area, and this proportion increased with the progress of the three waves. This suggested that the closure of LPMs led to development of alternative market channel options, including illegal trading [19, 20], or led urban families to purchase live poultry from rural markets. At least 8 urban cases were found to be infected by H7N9 virus through exposure to rural markets.

Our study showed that the proportions of positive towns and premises increased nearly 6 times from the first wave to the third. The expanding geographical range of contamination by H7N9 virus suggests that human infections will continuously emerge and may develop into new waves of epidemics. Compared to other avian influenza viruses, H7N9 virus is more adaptive to infection of mammals [1] and is readily transmitted through direct contact [21]. Data from the China Information System for Disease Control and Prevention identified 17 clustering events in China as of February 2015, and 11 of them might have been transmitted from person to person. Therefore, this implies that once H7N9 virus develops the ability for sustained transmission between humans, it may lead to a new round of pandemic influenza.

Our results also showed that the positive rates of H7N9 virus in sewage used for cleaning poultry and from swabs of tables used for slaughtering or processing poultry were significantly higher than in other environmental samples. This might be due to droplets generated during poultry slaughtering that contain viral particles [3]. The increasing positive rate of H7N9 virus from the first to the third wave suggested an enhancement of viability in the environment and transmissibility among poultry, which might be due to the change in its genetic architecture caused by co-circulation and reassortment between H7N9, H9N2 and other influenza viruses [22, 23].

Additional analysis found that the positive rate of H7N9 virus was significantly higher on premises that were positive for both H7 and H9 than on those that tested positive only for H7. Thus, regular phylogenetic analysis is needed to uncover the genetic variation and recombination of H7N9 virus. In the meantime, disinfection and other measures also should be taken to eliminate the rich environment for reassortment of these viruses.

As previous studies reported [3, 24], the positive rate of H7N9 virus in LPMs was much higher than in poultry rearing farms. In addition, we didn’t detect any positive samples in migratory bird habitats. These observations supported the following inferences on the sources and transmission of H7N9 virus: the virus is reassorted on poultry farms [6], and then reproduces, is transmitted and evolves mainly in LPMs owing to the markets’ ideal environment in China [25]. This suggests that more severe measures including disinfection, market rest periods and persistent surveillance should be conducted before all LPMs are permanently closed both in urban and rural area.

Of note, with the closure of LPMs in central towns, more and more poultry slaughtering and processing plants have been constructed as a new alternative to meet the new consumption pattern. There was a high positive rate of environmental specimens collected from these plants in the third wave, whereas no positive samples were identified in the past two waves. Therefore, similar measures, such as surveillance, disinfection and supervision, should also be strictly carried out in all slaughtering and processing plants to avoid H7N9 virus contaminating trade markets. We noticed that the positive rate at other environmental premises was also high, and the data showed that these samples were mostly collected from places where H7N9 cases lived. However, detailed investigation should be conducted to clarify the process of contamination.

In urban areas, attributable to closure of LPMs in central towns, the proportion of positive towns, premises and environmental samples were not significantly different for the second and third waves. As a matter of fact, the contamination area of H7N9 virus is expanding in urban areas because we did not count positive sites or specimens distributed in central towns in the third wave (after the LPMs were closed). Moreover, the proportion of positive towns, premises and environmental samples in rural areas increased rapidly from the first wave to the third, suggesting that strict control measures should be taken in rural areas as well.

Serological surveys of poultry-related workers showed a positive rate above 6 %, which was similar to what was reported in a previous study in Zhejiang Province [26] and significantly higher than in another southern province of China [3], where an H7N9 viral epidemic emerged later than in Zhejiang. More importantly, we found that the positive rate was much higher among workers from farms than from LPMs. Detailed investigation showed that these positive workers were concentrated in several villages, which might provide clues for tracing the source of H7N9 human infections.

Our study has some limitations. First, because the premises used for collection of environmental specimens were selected from an area with a higher risk of presence of H7N9 virus, the positive rates of H7N9 virus in the environment might be overestimated. However, the effect on the trend across the three waves was slight since the principles for selecting premises and sampling sites was unchanged. Secondly, serological surveillance was performed in the influenza epidemic period (the first quarter of each year), so the positive rate of poultry-related workers might be overestimated as well. Finally, the survey was conducted once a quarter in each prefecture in 2013. Although a third of counties were randomly selected each time, they can’t all be completely covered in the same season, which would decrease the accuracy of positive rates.

## Conclusion

In conclusion, our study highlights that poultry-related environmental contamination by H7N9 virus is intensifying. In light of our findings, we strongly recommend that the local government attach great importance to this serious issue and take effective measures immediately. Knowledge of safe methods of consumption of live poultry and related products must be forwarded to civilians to help them improve their habits of fresh poultry ingestion. Management of the live poultry trade should be more rigorous and illegal trading must be stopped immediately. If possible, local governments should close all LPMs (including wholesale and retail markets) in the whole territory, speed the construction of standard plants for slaughtering and processing live poultry, and establish a supply system for cold fresh poultry. Moreover, poultry operations in slaughtering and processing plants must be managed and supervised rigorously to avoid entry of contaminated cold fresh poultry to trading markets, supermarkets, restaurants and other related premises. Prior to the closure of LPMs, disinfection, rest days and other measures should be carried out strictly both in urban and in rural areas. These measures should include thorough daily cleaning and disinfecting of all areas and facilities potentially contaminated by H7N9 virus, centralized collection and proper disposal of trash and dead poultry, designating certain days as market rest days, and banning of overnight poultry storage.
